# Biodegradation of Tetracycline Antibiotics by the Yeast Strain *Cutaneotrichosporon dermatis* M503

**DOI:** 10.3390/microorganisms10030565

**Published:** 2022-03-05

**Authors:** Hao Tan, Delong Kong, Qingyun Ma, Qingqing Li, Yiqing Zhou, Xu Jiang, Zhiye Wang, Rebecca E. Parales, Zhiyong Ruan

**Affiliations:** 1 CAAS-CIAT Joint Laboratory in Advanced Technologies for Sustainable Agriculture, Institute of Agricultural Resources and Regional Planning, Chinese Academy of Agricultural Sciences, Beijing 100081, China; tanhao@caas.cn (H.T.); kongdelong009@163.com (D.K.); liqingqing@caas.cn (Q.L.); zhouyiqing@caas.cn (Y.Z.); jiangxu@caas.cn (X.J.); 2 Graduate School of Chinese Academy of Agricultural Sciences, Beijing 100081, China; 3 State Key Laboratory of Agricultural Microbiology, Huazhong Agricultural University, Wuhan 430070, China; mqy@webmail.hzau.edu.cn; 4 Key Laboratory of Microbial Resources Exploitation and Application of Gansu Province, Institute of Biology, Gansu Academy of Sciences, Lanzhou 730000, China; zhiye_wang@sina.com; 5 Department of Microbiology and Molecular Genetics, College of Biological Sciences, University of California, Davis, CA 95616, USA; reparales@ucdavis.edu; 6 College of Resources and Environment, Tibet Agricultural and Animal Husbandry University, Linzhi 851418, China; 7 College of Life Sciences, Yantai University, Yantai 264005, China

**Keywords:** tetracycline, biodegradation, *Cutaneotrichosporon dermatis*, response surface methodology (RSM), degradation products, antibacterial potency

## Abstract

In this study, the *Cutaneotrichosporon dermatis* strain M503 was isolated and could efficiently degrade tetracycline, doxycycline, and chlorotetracyline. The characteristics of tetracycline degradation were investigated under a broad range of cultural conditions. Response surface methodology (RSM) predicted that the highest degradation rate of tetracycline could be obtained under the following conditions: 39.69 °C, pH of 8.79, and inoculum dose of 4.0% (*v*/*v*, ~3.5 × 10^6^ cells/mL in the medium). In accordance with the five identified degradation products of tetracycline, two putative degradation pathways, which included the shedding of methyl and amino groups, were proposed. Moreover, the well diffusion method showed that the strain of M503 decreases the antibacterial potency of tetracycline, doxycycline, and chlorotetracycline. These findings proposed a putative mechanism of tetracycline degradation by a fungus strain and contributed to the estimation of the fate of tetracycline in the aquatic environment.

## 1. Introduction

Antibiotics play an extremely important role in the prevention and treatment of diseases caused by microorganisms and are widely used in humans and animals [1,2]. In 2013, the global consumption of veterinary antibiotics reached 131,109 tons, and it is expected to increase to 200,235 tons by 2030 [3]. Given the abuse of antibiotics and the stability of their structures, the accumulation of antibiotic residues inevitably occurs in the environment [4]. Antibiotic residues have been detected in aqueous (e.g., sewage, rivers, lakes, underground water, and drinking water) and terrestrial (e.g., sludge, sediments, and agricultural soil) environments [5,6,7,8,9,10,11]. These residues have selective effects on environmental microorganism, causing the proliferation of antibiotic resistance genes (ARGs), which adversely influence other organisms by inhibiting chlorophyll, protein synthesis and inducing oxidative stress [12,13,14,15].

Tetracycline antibiotics (TCs), including tetracycline (TC), doxycycline (DC), chlorotetracycline (CTC), are the most used veterinary drugs in the world [16,17]. Since the intestines of animals cannot completely absorb or metabolize TCs, about 30–90% of TCs are excreted into the environment through urine and feces [18,19,20]. Some studies even found high concentrations of TCs residues in manure (e.g., 78.6 mg/kg TC [21], 764.4 mg/kg CTC [22], and 78.5 mg/kg DC [23]). However, the removal efficiency of TCs through traditional treatments, such as compost and activated sludge, is sometimes unstably processed. Bao et al. (2009) found that the removal rate of CTC in hog manure is only 27% after 42 days of composting [24]. About 12% of TC, 35% of DC, and 28% of OTC in sewage are eliminated after being treated by a wastewater treatment system [4,25]. Other studies also used montmorillonite nanoclay and biochar to remove antibiotic contaminants [26,27]. 

Biodegradation is important in wastewater treatment of antibiotic contamination in the environment. Han et al. (2020) proved that anaerobic and aerobic biodegradation was an efficient way to remove veterinary antibiotics from swine wasterwater [28]. Existing studies proved that crude manganese peroxidase and lignin peroxidase extracted from *Phanerochaete chrysosporium* can efficiently eliminate TC and OTC, respectively [29,30]. The thermostable laccase from *Pycnoporus* sp. SYBC-L10 was also identified as completely removing TC and OTC [31]. However, the enzymatic reaction system requires diverse accessories, such as H_2_O_2_ and Mn^2+^, which increase the cost input for practical application. The toxicity of degradation products is also a concern after the removal of TCs. Becker et al. (2016) proposed that degradation products of TCs seem to generate unexpected toxicity during the removal of antibiotics by enzymatic treatment [32]. On the other hand, the toxicity of the degradation products of TCs by microorganisms decreases, e.g., *Trichosporon mycotoxinivorans* strain XPY-10 [33], *Stenotrophomonas maltophilia* DT1 [34], *Brevundimonas naejangsanensis* DD1, and *Sphingobacterium mizutaii* DD2 [35]. So, TCs degradation by microorganisms seems to be a safer method compared to enzymatic degradation. However, most studies are concerned about bacterial species, and knowledge about the TC degradation mechanism via the fungi strain is lacking.

In this study, the *C. dermatis* strain M503 is isolated as a TC-degrading fungus. Degradation characteristics are examined under different cultural conditions. Response surface methodology (RSM) is used to predict the maximum degradation rate of TC. Degradation products are identified, and potential degradation pathways are proposed. Moreover, the antibacterial potency of degradation products by strain M503 is determined. These findings may improve the understanding of TC degradation by fungal strains and the fate of TC in aquatic water.

## 2. Materials and Methods

### 2.1. Chemicals and Medium

TC hydrochloride (purity ≥ 96%), DC hydrochloride (purity ≥ 98%), and CTC hydrochloride (purity ≥ 80%) were purchased from Aladdin Industrial Co., Ltd. (Shanghai, China). HPLC-grade methanol and acetonitrile were purchased from MREDA Biotech Co., Ltd. (Beijing, China). The minimal mineral salt medium (MSM) consisted of 1.0 g/L NH_4_Cl, 1.0 g/L NaCl, 0.5 g/L KH_2_PO_4_, 1.5 g/L K_2_HPO_4_, 1.0 g/L CaCl_2_, and 0.2 g/L MgSO_4_·7H_2_O (pH 7.0). The PSM medium was made by adding 5 g/L potassium acetate to MSM. The lysogeny broth (LB) was composed of 10 g/L tryptone, 5 g/L yeast extract, and 10 g/L NaCl, and potato dextrose agar (PDA) was prepared in accordance with the method of Song et al. (2013) [36]. After the tetracycline powder was dissolved in deionized water and filtered by 0.22 μm sterile membrane, the TC solution (5 g/L) was prepared and placed in 4 °C refrigerator for further experiments.

### 2.2. Enrichment and Isolation of TC-Degrading Strains

The contaminated sediment was collected from sediment tank of sewage treatment system at a TCs manufacturing company in Lanzhou, Gansu Province, China. The enrichment process was conducted by adding 5 g samples into MSM containing 50 mg/L TC and incubating at 30 °C in a rotary shaker at 150 rpm. Seven days later, 1 mL solution was serially diluted and coated on MSM plates with 50 mg/L TC. Plates were incubated at 30 °C for four days, and different colonies were purified by streaking thrice onto LB plates. After two days of incubation in LB at 30 °C in a rotary shaker, the pellets of all isolates were washed twice and inoculated in PSM medium containing 50 mg/L TC. Conical flasks were wrapped by foil and incubated in a rotary shaker at 30 °C and 150 rpm, and the TC concentration was monitored by HPLC.

### 2.3. Identification of Strain M503

Among four TC-degrading isolates, the strain M503 showed the highest TC degradation rate and was selected for further study. The purified strain M503 was inoculated onto a PDA plate, and the phenotypic characteristics were described after incubation at 30 °C for seven days. The DNA extraction of strain M503 was carried out in accordance with the instructions of the Fungal DNA Extraction Kit (Solarbio, Beijing, China). The amplification of ITS sequence with ITS primers ITS1 (5′-TCCGTAGGTGAACCTGCGG-3′) and ITS4 (5′-GCATATCAATAAGCGGAGGA-3′) was performed on the basis of the extracted DNA, and PCR program following the PCR protocol described by Montoya et al. (2020) [37]. Sequencing was implemented by Sangon Biotech Co., Ltd., China, and BLAST was used to compare known strains and sequencing results. Phylogenetic analysis was conducted as described in a previous study [38].

### 2.4. Optimization of Cultural Conditions for C. dermatis M503

The LB medium inoculated with strain M503 was incubated for two days in a rotary shaker at 30 °C and 150 rpm. Pellets were collected after centrifugation for 10 min at 8000 rpm and washed twice with sterile water, the OD_600_ of the suspension was adjusted to 1.00 (8.7 × 10^7^ cells/mL). All experimental groups were inoculated with 5.0% (*v*/*v*, ~4.35 × 10^6^ cells/mL in the medium) strain M503 and incubated in a rotary shaker at 30 °C and 150 rpm. Five series of experiments were conducted for the optimization of cultural conditions:

(1) Six different carbon sources (potassium acetate, glucose, sucrose, maltose, and peptone, all at 5 g/L) at 30 °C and initial pH of 7.0;
(2) Five concentrations of potassium acetate (1, 2.5, 5, 10, and 15 g/L) at 30 °C and initial pH of 7.0;
(3) Five incubation temperatures (20 °C, 25 °C, 30 °C, 35 °C, and 40 °C) at initial pH of 7.0;
(4) Five initial pH (6, 7, 8, 9, and 10) at 30 °C;
(5) Five initial TC concentrations (50, 100, 150, 200, and 300 mg/L) at 30 °C and pH of 7.0.

For the five groups of experiments, (1) all experiments were conducted in triplicate; (2) all experimental initial TC concentrations were 100 mg/L except for experiment (5); (3) all vials were wrapped with aluminum foil and incubated in the dark; and (4) sampling was performed continuously for seven days.

### 2.5. Experiments on Biodegradation Property of Strain M503 

#### 2.5.1. Response Surface Methodology for Optimal TC Degradation Rate

Three factors were considered to affect TC degradation. On the basis of the Box–Behnken design, experiments with 3 factors at 3 levels (−1, 0, +1), i.e., incubation temperature (20 °C, 30 °C, and 40 °C), initial pH (5, 7, and 9), and inoculation doses (1%, 5.5%, and 10%), were conducted with 17 runs (Table S1). The samples of 17 runs were collected and tested at 0 d and 5 d. The second-order polynomial equation described by Ruan et al. (2013) [39] was used.

#### 2.5.2. Identification of TC Degradation Products of Strain M503

Solutions were collected at seven days from two experimental groups: (1) PSM medium containing 100 mg/L TC with strain M503 and (2) PSM medium containing 100 mg/L TC. Then, the solutions were centrifuged at 10,000 rpm for 10 min. Solid-phase extraction cartridges (Oasis HLB, 6 cc/150 mg, Waters) were used to extract TC and products in the solutions as described by Leng et al. (2016). A UPLC coupled with the Q-TOF-MS (Acquity, Bruker, Bremen, Germany) was used for the analysis of TC degradation products. The conditions of separation and detection were consistent with the previous study [40]. The mass spectral data were processed by MassLynx (Waters, Milford, MA, USA, version 4.1), and molecular constructions were conducted by Kingdraw (version 2.1).

#### 2.5.3. Quantification of TC, DC, and CTC

After sampling 3 mL solutions of each group, centrifugation was conducted to remove pellets, and the supernatant was filtered by 0.22 μm nylon filters. Then, the supernatant was performed on the Agilent 1260 system with a C_18_ column (4.6 × 100 mm, 3.5 μm). The eluents consisted of 8% acetonitrile, 8% methanol, and 84% ultrapure water containing 1% acetic acid. The HPLC elution conditions were as follows: injection volume of 20 μL, column temperature of 30 °C, flow rate of 1.0 mL/min, and UV wavelength of 355 nm. Considering that TCs had similar molecular structures, the biodegradability of DC and CTC was also investigated. The PSM media with 100 mg/L DC and CTC were prepared, respectively. After supplementing with strain M503 (5% (*v*/*v*)), the medium was incubated at 30 °C in a rotary shaker of 150 rpm. Both residues were detected by the Agilent 1260 system equipped with a C_18_ column. The detection conditions of DC were as described by He et al. (2021), and CTC was detected using a mixed solution of 1% acetic acid:acetonitrile:methanol (77:18:5 (*v*/*v*/*v*)) under a flow rate of 1.0 mL/min, an injection volume of 60 μL, temperature of 40 °C, and UV wavelength of 350 nm.

### 2.6. Antibacterial Potency of the Degradation Products of TC, DC, and CTC

Two sets of experiments were evaluated: (1) experimental group (M503)—PSM solution with 100 mg/L of TC containing 5% (*v*/*v*) strain M503, and (2) negative control (CK)— PSM solution with 100 mg/L of TC containing inoculation of 5% (*v*/*v*) sterile water. The same experiments were conducted on DC and CTC. The well diffusion method was adopted to determine the antibacterial potency of TC, DC, CTC, and their degradation products [37]. *Escherichia coli* ATCC 25922 was selected as indicator strain and grown in LB medium at 30 °C to a steady phase. Pellets were collected from an LB medium and diluted to OD_600_ = 1.00, and 100 μL of solution was coated onto LB agar plates, then Oxford cups (diameter = 7.8 mm) were placed on LB agar plates. To test the antibacterial potency of TC and its degradation products, cups were filled with 200 μL medium filtered by 0.22 μm sterile membrane, which were collected from various phases of the experiments with strain M503 (M503) and control experiments without strain M503 (CK). The same operations were conducted with 100 μL medium to DC and CTC. The diameter of inhibition zones (including the diameter of cup) was measured after incubation at 30 °C for 20 h.

## 3. Results

### 3.1. Isolation and Identification of Strain M503

**Figure 1:**
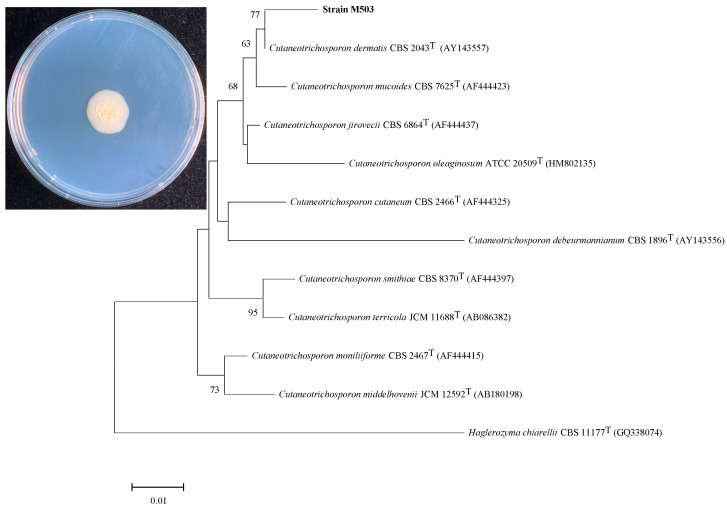
Strain M503 colony morphology on PDA at 30 °C for seven days. Phylogenetic tree on the basis of the ITS gene sequences (OM108212) of *C. dermatis* and other related strains. GenBank accession numbers are in parentheses.

Four isolates were obtained from MSM agar plates containing 50 mg/L TC and labeled M501–M504. Since strain M503 had the highest degradation rate (61.24% at day 2) under 50 mg/L of TC, it was selected for further analysis. Strain M503 was a yeast strain and characterized by white colonies with a centrally cerebriform, smooth, and radical fissure surface on PDA (Figure 1). Strain M503 could grow in PSM medium with 100 mg/L TC over a temperature range of 20 °C–40 °C and pH range of 6.0–10.0.

Based on the ITS gene sequence, the phylogenetic analysis revealed that strain M503 was identified as belonging to the *Cutaneotrichosporon* group and showed the highest similarity with *Cutaneotrichosporon dermatis* (99.85%). According to morphological characteristics and phylogenetic tree, strain M503 was identified as *C. dermatis* species. Strain M503 is the first strain of *C. dermatis* reported to degrade TC.

### 3.2. Effects of Different Carbon Sources and Potassium Acetate Concentrations

**Figure 2:**
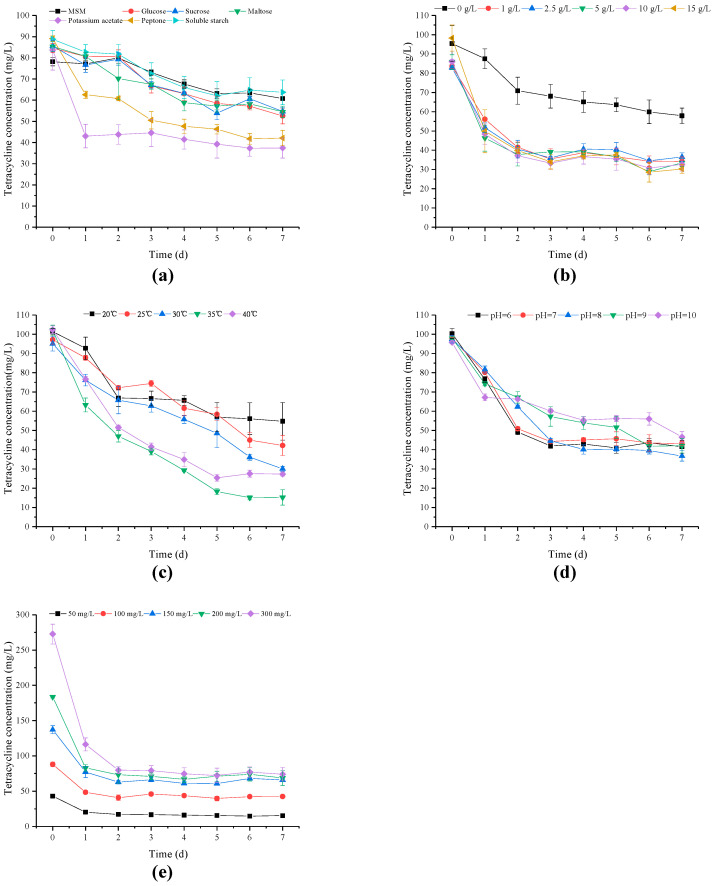
Effects of different carbon sources (**a**), potassium acetate concentrations (**b**), temperature (**c**), pH values (**d**), and initial tetracycline concentrations (**e**) on TC degradation by strain M503. Values are expressed as mean of three replicates with standard deviations.

The TC degradation of strain M503 was carried out with different carbon sources, including glucose, sucrose, maltose, peptone, potassium acetate, and soluble starch. Figure 2a shows the corresponding TC degradation curves. Among the six carbon sources, the potassium acetate showed the highest degradation rates of TC, whereas the soluble starch group had the lowest TC degradation. Compared with the control group, all carbon source groups, except the soluble starch group, had lower residual TC concentrations. The TC degradation rate of the peptone group reached 52.70%, which indicated that peptone could be the supplementary carbon source for microorganisms to remove TC [34,35]. Considering that the TC degradation rate of the potassium acetate group was higher than that of the peptone group, potassium acetate was chosen for subsequent experiments. 

The influence of potassium acetate concentration on degradation efficiency is shown in Figure 2b. The effects of different potassium acetate concentrations on the degradation of TC followed the order of 15 g/L > 10 g/L > 5 g/L > 1 g/L > 2.5 g/L > 0 g/L, exhibiting TC degradation values of 69.23%, 62.07%, 60.97%, 59.07%, 55.97%, and 39.31%, respectively. The results demonstrated that the TC degradation rate increased with the supplemental mass of potassium acetate because the strain M503 had increased primary carbon to propagate and degrade TC. During the application of microorganisms in TC wastewater treatment, minimal input is preferred [41]. Overall, to obtain a higher degradation rate of TC and biomass, whilst decreasing the input, a concentration of 5 g/L was required for a proper concentration of potassium acetate.

### 3.3. Effects of Temperature, pH, and Initial TC Concentration

Temperature is a significant factor for TC degradation in aquatic environments [42,43]. Heat-labile TC is found by Wu et al., (2005). TC shows different heat stability values in different substrates, and hydrolysis rates increase with increased temperature [44,45]. The degradation differences caused by temperature are presented in Figure 2c. The order of TC degradation rates was as follows: 35 °C > 40 °C > 30 °C > 25 °C > 20 °C. The degradation rate increased with increased temperature. The highest degradation of TC (85.10%) was found at 35 °C, implying that 35 °C was the optimal temperature of strain M503 when the initial pH value was 7.

The pH of the aqueous solution is a critical factor in TC stability [42]. As shown in Figure 2d, when the initial pH increased from 6 to 10, TC degradation rates of 58.61%, 55.74%, 62.32%, 57.09%, and 51.43% were obtained within seven days. No remarkable difference was observed in the range of 6–10, indicating that the strain M503 had good adaptability to environmental pH.

The effects of initial TC concentration (50 mg/L to 300 mg/L) are presented in Figure 2e. The strain M503 had a high tolerance to TC even at the concentration of 300 mg/L and expressed a high TC degradation rate (73.55%). This finding confirmed that strain M503 had the potential for the remediation of environmental contamination even with a high TC content.

### 3.4. RSM Analysis of TC Degradation by Strain M503

The RSM based on the Box–Behnken design was performed to determine the influences of cultural conditions on the TC degradation of strain M503. Table S1 shows the matrix of design and the value of TC degradation. On the basis of these data, the Design-Expert (8.0.6) with the SAS software package was used to predict the responses of TC degradation to cultural conditions, including incubation temperature, medium pH, and inoculum doses. The second-degree polynomial equation was expressed as follows:

Y_M503_ = 56.66 + 19.90X_1_ + 14.13X_2_ + 5.77X_3_ + 8.21X_1_X_2_ − 8.02X_1_X_3_ − 8.48X_2_X_3_ − 7.27X_1_^2^ − 8.47X_2_^2^ + 1.60X_3_^2^, (1)

where Y_M503_ is the predicted value of response TC degradation, and X_1_, X_2_, and X_3_ are coded values of incubation temperature, medium pH, and initial inoculum doses, respectively. The second-degree polynomial equation model was significant (*p* < 0.05, R^2^ = 0.9846). The regression analysis indicated that incubation temperature (X_1_), pH (X_2_), inoculum dose (X_3_), and interaction terms were significant (*p* < 0.05), whereas the square term of inoculum dose (X_3_) was not significant (*p* > 0.05).

**Figure 3:**
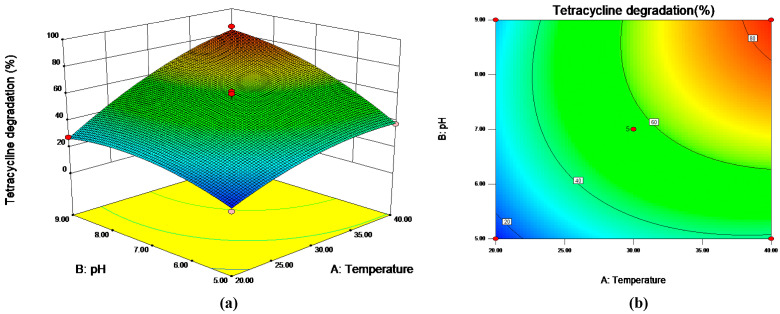
Response 3D surface (**a**) and contour plot (**b**) for the change in tetracycline degradation under different temperatures and pH values with fixed inoculum volume of 5.5% (*v*/*v*).

As presented in Figure 3b, the results of the contour plot showed that high TC degradation was obtained at around pH 8 when the incubation temperature was fixed. The TC degradation increased with increasing temperature from 20 °C to 40 °C. These findings were consistent with those observed in the optimization of medium pH and incubation temperature. The theoretical maximum TC degradation rate predicted by RSM was 86.62%, and the optimal cultural conditions (Figure 3a) were 39.69 °C, pH of 8.79, and inoculum dose of 4.0%. 

### 3.5. TC Degradation Products

**Figure 4:**
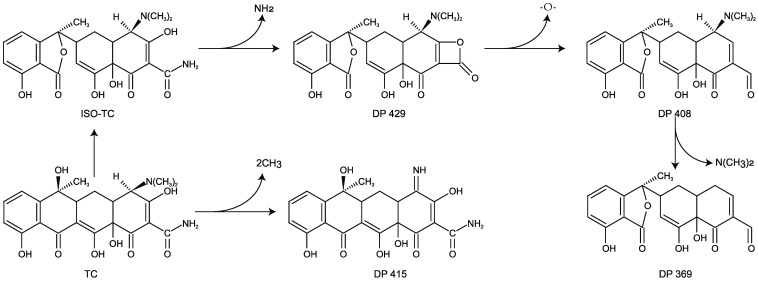
Putative degradation pathways of TC by strain M503.

As shown in Figure S1, the results of mass spectrum identified five degradation products (DP), such as DP 429, DP 415, and DP 408. Two degradation pathways of TC by strain M503 are proposed in Figure 4. In the first degradation pathway, two N-methyl groups dropped out from the parent compound TC (*m*/*z* = 445) and transformed into compound 415. In the second degradation pathway, TC was converted into ISO-TC (isomer of TC), and then compound 429 was generated after losing an amino group from the compound ISO-TC. When a breakage occurred at the ether bond, compound 408 was formed. Finally, the groups of N-methyl and amino separated from compound 408 and formed compound 369.

### 3.6. Degradation of TC, DC, and CTC by Strain M503 and the Antibacterial Potency of Degradation Products

**Figure 5:**
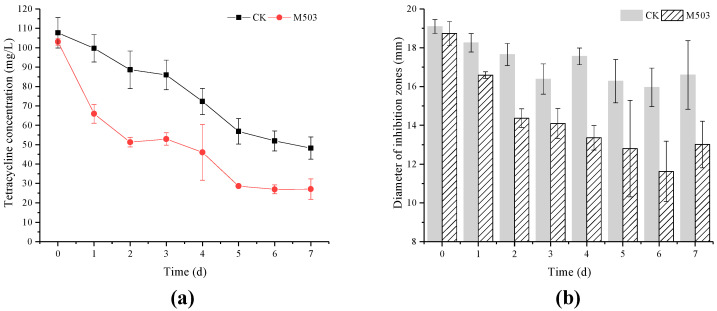
Tetracycline degradation curves (red, M503: group with strain M503; black, CK: group without strain M503) (**a**). Antibacterial potency of the degradation products of two groups (**b**). Values are expressed as mean of three replicates with standard deviations.

The antibacterial potency of TC and its degradation products by strain M503 was lower than the control group (Figure 5). For the treatment group, the TC residues decreased from 103.07 mg/L to 27.07 mg/L within seven days, and the diameters of inhibition zones decreased from 18.73 mm to 13.01 mm. In the control group, the residual TC dropped from 107.65 mg/L to 48.24 mg/L, and the inhibition zones decreased from 19.09 mm to 16.59 mm. 

**Figure 6:**
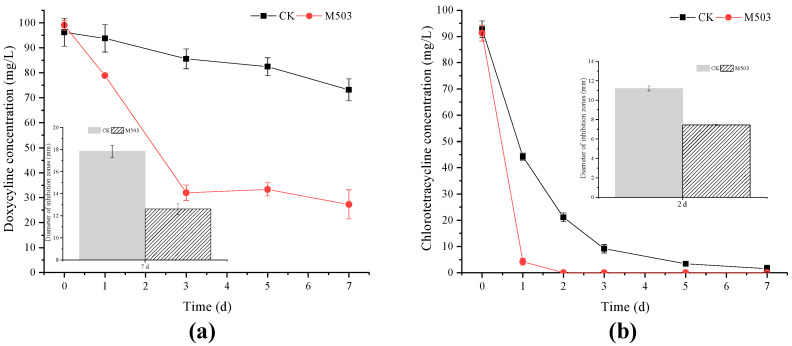
Doxycycline degradation curves and antibacterial potency of degradation products of two groups at seven days (bar plot) (**a**). Chlorotetracycline degradation curves and antibacterial potency of degradation products of two groups at two days (bar plot) (**b**). (M503: group with strain M503; CK: group without srain M503.) Values are expressed as mean of three replicates with standard deviations.

The biodegradability of strain M503 to DC and CTC was evaluated (Figure 6). After inoculation with strain M503, the residues of DC dropped from 99.09 mg/L to 27.25 mg/L within seven days, and this value was about 72.50% of the DC degradation rate and higher than that of the control (23.92%). The diameter of the inhibition zones of the experimental group was 12.61 mm at day 7, which was lower than that of the control group (17.83 mm).

In the experiments of CTC degradation, the strain M503 could degrade 100 mg/L CTC completely within two days. The differences in the inhibition zones between the experimental and control groups indicated that the biodegradability of strain M503 could alleviate the antibacterial potency of TC, DC, and CTC.

## 4. Discussion

Strain M503 was isolated as a TC-degrading fungus species and could use additional carbon sources to degrade a high concentration of TC. These findings were consistent with those of previous studies [35,46], and potassium acetate is a better choice than peptone for strain M503. Given their complex molecular structure, TCs are regarded as the sole carbon source for growth of only few strains, e.g., strain of *Pseudomonas* sp. T4 and *Pandoraea* sp. TJ3 [47,48]. In this study, the results proposed that co-metabolism is an effective way to eliminate obstinate pollutants in the environment.

As shown in Figure 2a,b, compared to the no-added-carbon-sources group (0 g/L group in Figure 2b), not all additional carbon sources contributed to the degradation of TC, such as soluble starch, which could provide sufficiently rich carbon sources to meet the requirements of microbial growth, so that strain M503 did not have to degrade the obstinate TC. Previous studies obtained similar results [47]. The results of optimization experiments showed that strain M503 expressed a high degradation rate at a temperature range of 20 °C–40 °C. The highest degradation rate occurred at 35 °C, which was a higher temperature than some bacterial strains, such as *S. maltophilia* DT1, *A. nicotianae* OTC-16, *B. naejangsanensis* DD1, and *S. mizutaii* DD2 [34,35,49]. The difference in optimal temperature between bacteria and strain M503 showed that strain M503 might prefer a higher temperature to perform degradation functions. As shown in Figure 2d, strain M503 performed well in TC removal at a pH range of 6–10, and the optimal initial pH value was 8. The results were slightly different with some reported strains, such as, *S. maltophilia* DT1, *Klebsiella* sp. SQY5, but similar to *S. mizutaii* DD2. Since the wastewater of pharmaceutical industry expressed a high value of pH [50], the good performance in TC degradation of strain M503 in alkaline conditions indicated that it could be a potential bioresource for the treatment of pharmaceutical wastewater. In the experiments of initial TC concentration ranging from 50 mg/L to 300 mg/L (Figure 2e), the highest degradation rate (73.55%) was obtained in the group with 300 mg/L TC. The high degradation rates at high concentrations of TC were rarely found in the studies of bacterial strains.

The putative degradation pathways of TC by strain M503 showed that huge differences existed in the metabolism of fungi and bacteria. Although, compound 415 and ISO-TC are detected by a few studies on TC degradation by bacteria such as *S. maltophilia* DT1 and *S. Changzhouense* TC931 [46], other products, such as compounds 408 and 369, were rarely identified. The different degradation products between strain M503 and bacteria could be ascribed to the diverse degrading enzymes or metabolic pathways, which needed to be further studied. The identification of compound 415 in several studies indicated that de-methylation at N-(CH_3_)_2_ group might be the essential process for degrading TC. The deamination reaction was also found in the degradation pathway of TC by strain *S. maltophilia* DT1 and *Klebsiella* sp. SQY5 [51]. These results proved that similar reactions existed in the degradation of TC by bacteria and fungi. Compound 408 was rarely identified by previous studies except for the degradation of TC by a yeast strain XPY-10 [33]. Compared to bacteria, yeast strains might have some unique reactions in the degradation of TC, such as the cleavage of the ether bond, so that the unique degradation product, compound 408, was obtained.

In the well diffusion experiments, higher concentrations of TC generally led to larger inhibition zones. However, the inhibition zones of experiments with strain M503 were smaller than control groups; even the concentration of TC in experimental group (M503) at day 2 was similar with it in control group (CK) at day 7. This is evidence that the antibacterial potency of TC degradation products by strain M503 decreased. Similarly, many studies found that the biotransformation products of TCs have a lower antibacterial potency [35,49]. This finding is an indication that microorganisms have the intellective mechanism to decrease the toxicity of antibiotics to bacteria. Nevertheless, the assessment of antibacterial potency was performed using experimental solutions and was not an accurate exhibition of TC degradation products. Hence, the scientific standard to evaluate the antibacterial potency of TC degradation products is urgently acquired.

## 5. Conclusions

This study confirmed for the first time that *C. dertamis* species can not only degrade TC, DC, and CTC, but can also decrease the antibacterial potency of them. The RSM analysis predicted that the highest degradation rate of TC (86.62%) can be obtained under the conditions of 39.69 °C, pH of 8.79, and inoculum dose of 4.0% (~3.5 × 10^6^ cells/mL in the medium). In addition, a unique fungal degradation pathway was proposed based on the results of mass spectrum. This study demonstrated that strain M503 can degrade TC, DC, and CTC efficiently, which provides a potential bioresource for bioremediation of TCs-contaminated soil or wastewater.

## Data Availability

Not applicable.

## Supplementary Materials

The following are available online at https://www.mdpi.com/article/10.3390/microorganisms10030565/s1, Figure S1: Mass spectrum of five TC degradation products; Table S1: Box-Behnken design of strain M503 and responses.
