# Supplementary material for: Biodegradation of Tetracycline Antibiotics by the Yeast Strain Cutaneotrichosporon dermatis M503
Source: Microorganisms. 2022 Mar 5;10(3):565. doi: 10.3390/microorganisms10030565 (PMC8955161; doi:10.3390/microorganisms10030565)
Supplement: Supplementary file 1 [file microorganisms-10-00565-s001.zip › microorganisms-1598409-supplementary.pdf]

Supporting materials

Captions

Table S1 Box-Behnken design of strain M503 and responses

Figure S1 Mass spectrum of five TC degradation products.

Table S1 Box-Behnken design of strain M503 and responses

Run	X_1	X_2	X_3	Response tetracycline degradation (%)
1	0	0	0	52.85±1.19
2	-1	-1	0	12.64±0.38
3	0	0	0	54.37±0.72
4	-1	1	0	27.59±0.51
5	-1	0	-1	18.47±1.91
6	-1	0	1	45.47±1.31
7	1	-1	0	37.8±0.21
8	0	-1	1	51.75±0.37
9	1	0	-1	72.53±0.57
10	1	0	1	67.45±0.30
11	0	1	1	59.95±0.30
12	0	-1	-1	22.66±0.76
13	0	0	0	53.53±1.24
14	0	1	-1	64.77±0.92
15	0	0	0	60.36±0.92
16	0	0	0	62.17±0.92
17	1	1	0	85.61±1.84

X_1: temperature, -1 (20 ˚C), 0 (30 ˚C), 1 (40 ˚C); X_2: pH, -1 (5), 0 (7), 1 (9); X_3: inoculum, -1 (1%), 0 (5.5%), 1 (10%).

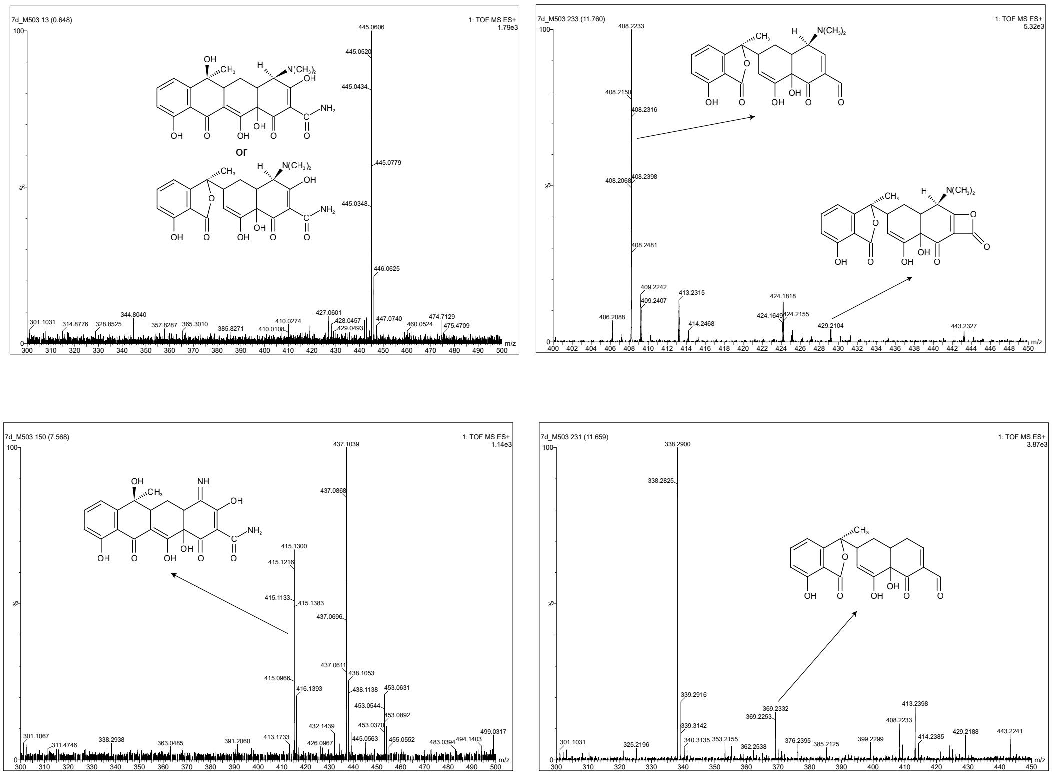

Figure S1 Mass spectrum of five TC degradation products.
